# Scaffolds in Tendon Tissue Engineering

**DOI:** 10.1155/2012/517165

**Published:** 2011-12-11

**Authors:** Umile Giuseppe Longo, Alfredo Lamberti, Stefano Petrillo, Nicola Maffulli, Vincenzo Denaro

**Affiliations:** ^1^Department of Orthopaedic and Trauma Surgery, Campus Bio-Medico University, Via Alvaro del Portillo, 200, 00128 Rome, Italy; ^2^Centre for Sports and Exercise Medicine, Barts and The London School of Medicine and Dentistry, Queen Mary University of London, Mile End Campus, 275 Bancroft Road, London E1 4DG, UK

## Abstract

Tissue engineering techniques using novel scaffold materials offer potential alternatives for managing tendon disorders. Tissue engineering strategies to improve tendon repair healing include the use of scaffolds, growth factors, cell seeding, or a combination of these approaches. Scaffolds have been the most common strategy investigated to date. Available scaffolds for tendon repair include both biological scaffolds, obtained from mammalian tissues, and synthetic scaffolds, manufactured from chemical compounds. Preliminary studies support the idea that scaffolds can provide an alternative for tendon augmentation with an enormous therapeutic potential. However, available data are lacking to allow definitive conclusion on the use of scaffolds for tendon augmentation. We review the current basic science and clinical understanding in the field of scaffolds and tissue engineering for tendon repair.

## 1. Introduction

Tissue engineering techniques using novel scaffold materials offer potential alternatives for managing tendon disorders [1]. Tissue engineering strategies to improve tendon repair healing include the use of scaffolds, growth factors, cell seeding, or a combination of these approaches [1]. Scaffolds have been the most common strategy investigated to date [1]. The rationale for using a scaffold device for tendon repair may include mechanical augmentation, improving the rate and quality of biologic healing, or both [2]. Scaffolds with robust mechanical and suture retention properties, applied in a surgically appropriate manner, may have the ability to “off-load” the repair at time zero and for some period of postoperative healing, depending on the rate and extent of scaffold remodeling. Despite the growing clinical use of scaffold devices for tendon repair, there are numerous questions related to their indication, surgical application, safety, mechanism of action, and efficacy that remain to be clarified or addressed [1].

The use of scaffolds alone in flexor tendons has not been highly studied, but they have been combined with tenocytes in an effort to engineer an autologous tendon graft [3, 4]. However, the use of scaffolds in flexor tendon repairs may have a detrimental effect on tendon gliding, due to their size, and the lack of space within a repaired synovial sheath.

Scaffolds for both the Achilles tendon and the rotator cuff have been investigated both as structural supports and as delivery systems for other tissue engineering modalities. Available scaffolds for tendon repair include both biological and synthetic scaffolds [5]. In this paper we review the current basic science and clinical understanding of scaffolds and tissue engineering for tendon repair. We underline benefits and limitations of the available scaffolds for augmentation of tendon disorders and discuss the implications of these data on future directions for the use of these scaffolds in tendon repair procedures.

## 2. Biological Scaffolds

Biological scaffolds are obtained from mammalian (human, porcine, bovine, and equine) tissues [6]. To remove any noncollagen components, thus, minimizing the risk of host rejection while retaining its natural collagen structure and mechanical properties, small intestine submucosa (SIS), dermis, and pericardium are processed through cascade steps, including general cleaning, removal of lipids or fat deposits, disruption of cellular and DNA materials, crosslinking, and sterilization [6]. The final scaffolds are composed mainly of naturally occurring collagen fibres, predominantly type I collagen, and several of them have a surface chemistry and native structure that is bioactive and promotes cellular proliferation and tissue ingrowth [6].

The Restore graft (Depuy, Warsaw, IN) is a circular implant consisting of 10 not crosslinked layers of porcine small intestinal submucosa (SIS). It is more than 90% collagen with approximately 5–10% lipids and a small amount of carbohydrate [7, 8]. Iannotti et al. [9] tried to determine the effectiveness of porcine SIS to augment the repair of rotator cuff. They randomized 30 shoulders with a chronic two-tendon rotator cuff tear (9 with a large tear and 21 with a massive tear of rotator cuff) that was completely repairable with open surgery to be managed with either augmentation with porcine SIS or no augmentation. The rotator cuff healed in 4 of the 15 shoulders in the augmentation group compared with 9 of the 15 in the control group. The authors concluded that augmentation of the surgical repair of large and massive chronic rotator cuff tears with porcine SIS did not improve the rate of tendon healing or the clinical outcome scores. On the basis of their investigation, they do not recommend using porcine SIS to augment repairs of massive chronic rotator cuff tears performed with the surgical and postoperative procedures described in this study. Metcalf et al. [10] conducted a 2-year followup of 12 patients who underwent arthroscopic repair of massive chronic rotator cuff tears using Restore SIS as an augmentation device. Postoperative magnetic resonance imaging (MRI) scans showed significant thickening of the cuff tendon with the incorporation of the SIS graft in 11 patients. In 1 of 12 patients, clinical failure was observed within 12 weeks with complete resorption of the graft. There was no evidence of local or systemic rejection or infection in any patient. The mean postoperative University of California, Los Angeles, (UCLA) score was 19.9 on a scale of 35, a significant improvement over the preoperative score of 9.9, but the shoulder function remained far below normal in these patients. This study demonstrated improved post-operative outcomes for patients managed with the Restore graft augmentation compared with their preoperative condition. However, the lack of a control group makes it difficult to conclude that the functional improvements in the study were the result of SIS augmentation. Sclamberg et al. [11] evaluated clinical and MRI at 6 months in 11 patients undergoing open repair of large or massive rotator cuff tears augmented with Restore. MRI showed a retear in 10 of 11 patients. Zheng et al. [12] performed a study to evaluate the safety and efficacy of the Restore SIS membrane. The Restore orthobiologic implant was examined by histology and the nested PCR technique using porcine immunoreceptor DAP12 gene to examine if SIS membrane contained porcine cells or DNA, respectively. The material was also implanted into mice and rabbits for the evaluation of biological reaction and inflammatory response. Restore SIS was found to contain multiple layers of porcine cells. Chloroacetate esterase staining showed that some of these cells were mast cells. Nested PCR of the DAP12 gene demonstrated that Restore SIS contained porcine DNA material. Subcutaneous implantation of Restore SIS membrane in mice, and in rabbits for rotator cuff tendon repair, showed that the membrane caused an inflammatory reaction characterized by massive lymphocyte infiltration. The authors concluded that Restore SIS is not an acellular collagenous matrix, and contains porcine DNA, contradicting the current view that Restore SIS is a cell-free biomaterial and that no inflammatory response is elicited by its implantation. Walton et al. [13] compared a group of patients who had undergone rotator cuff repair with xenograft augmentation with a group repaired without augmentation. Four patients in the xenograft group showed a severe postoperative reaction requiring surgical treatment. Two years postoperatively, MRI documented retears in 6 of the 10 tendons repaired with a xenograft and in 7 of the 12 nonaugmented tendons; the patients with a xenograft also had less strength than the controls and had more impingement in external rotation, a slower rate of resolution of pain during activities, more difficulty with hand-behind-the-back activities, and a lower rate sports participation.

The Zimmer Collagen Repair Patch (Tissue Science Laboratories, Covington, Ga, USA, licensed to Zimmer) is a single layer porcine skin xenograft. It is an acellular crosslinked collagen sheet of crosslinked porcine dermis. Soler et al. [14] used Zimmer Collagen Patch as a bridging device to repair massive rotator cuff tears. After a good postoperative period, between 3 and 6 months, the graft began to fail, and the patients showed signs and symptoms of retear, with also signs of inflammation. MRI scans showed inflammatory changes, resorption of the graft, fluid pooling in the subdeltoid bursa, and loss of continuity of the remaining graft material. Histology of the debris revealed necrotic fibrinous material on a background of chronic inflammation. Badhe et al. [15] prospectively evaluated 10 patients with extensive rotator cuff tear treated with Zimmer Collagen Patch (Permacol). All patients experienced significant pain relief and improvement in abduction power and range of motion. Ultrasound imaging at the final followup identified intact grafts in eight and disrupted grafts in two patients.

GraftJacket (Wright Medical Technology, Inc.) is a commercially available acellular dermal matrix obtained from tissue bank human skin, composed of collagen types I, III, IV, and VII, elastin, chondroitin sulfate, proteoglycans, and fibroblast growth factor. It has an intact basement membrane complex and preserved vascular channels to allow rapid infiltration of fibroblasts and vascular tissue, with minimal host inflammatory response [3, 8, 13]. Barber et al. [16] compared the failure mode of supraspinatus tendon repair with and without GraftJacket augmentation in a human cadaveric model. No significant displacement occurred during the cyclic phase, and no anchors failed. During the destructive testing phase, the mean load-to-failure strength of the control construct was 273 + 116 N. The load-to-failure strength of the supraspinatus tendon augmented with GraftJacket was 325 + 74 N. The constructs failed by two different mechanisms: tendon-suture interface failure (8/10 nonaugmented repairs and 6/10 augmented repairs) and suture breakage (2/10 non-augmented repairs and 4/10 augmented repairs). Bond et al. [17] treated 16 patients with massive rotator cuff tears with arthroscopic implantation of a GraftJacket allograft. At a mean followup of 26.7 months, 15 of 16 patients were satisfied with the procedure. The mean UCLA score increased from 18.4 preoperatively to 30.4 postoperatively. The mean pain score improved from 4.6 to 9.8 postoperatively. The mean constant score increased from 53.8 to 84.0. Statistically significant improvements were noted in pain, forward flexion, and external rotation strength. MRI scans showed full incorporation of the graft into the native tissue in 13 patients. Chronic Achilles tendon rupture, repaired with GraftJacket, showed early return to activity and good plantarflexion strength [18]. Two studies [19, 20] evaluated GraftJacket as an augmentation device in the Achilles tendon repair. In the first study [19], nine patients with chronic Achilles tendon ruptures were followed up. There were no reruptures or recurrent pain at 20–30 months postoperatively, and the average return-to-activity time was 15.2 + 1.7 weeks. In the second study [20], 11 patients with acute tendon ruptures were followed up for 20 to 31 months. At 20 months, there were no reruptures or recurrent pain; the average return-to-activity time was 11.8 + 0.75 weeks. Significant increase in strength and stiffness of Achilles tendon repair augmented with GraftJacket was also observed in a human cadaver model (12.99 + 5.34 N/mm versus 4.29 + 0.83 N/mm of the control group) [21].

## 3. Synthetic Scaffolds

Because allograft materials may cause inflammatory responses in the host, there is notable interest in developing synthetic extracellular matrix (ECM) grafts for surgical use. Synthetic ECMs may still serve as an adequate scaffold for cellular and fibrotic growth, while running a smaller risk of provoking an inflammatory response than allograft ECMs. Several animal studies have investigated the benefit of augmenting rotator cuff repair with synthetic ECMs.

Yokoya et al. [22] used a polyglycolic acid (PGA) sheet to augment rotator cuff repairs of infraspinatus tendons in Japanese white rabbits, showing histological improvement in fibrocartilage layering but only a slight improvement in tensile strength when compared to control tendons augmented with another slowly absorbing synthetic material [22]. In a similar study, Funakoshi et al. [23] demonstrated increased fibroblast presence and collagen formation when synthetic ECM was surgically applied to rotator cuff tears. In this experiment, a 10-mm surgical defect was created at the humeral insertion of the infraspinatus tendon in 21 Japanese white rabbits. In one shoulder, the 10-mm defect was covered with chitin, a biodegradable polymer, sutured into the bone trough, and attached to the free end of the infraspinatus tendon. The contralateral shoulder was left untreated as a control. Throughout the experiment, tendon-to-bone junctions covered with chitin fabric demonstrated greater cell number, better collagen fiber alignment, and greater mechanical strength than the tendon-to-bone junctions left free as control [23]. In another study, MacGillivray et al. [24] used polylactic acid patches in goats, showing no observable difference between the treated and control groups [24]. A similar experiment, using a woven poly-L-lactide device, was performed by Derwin et al. [25] in a dog model. The superior 2.3 of each infraspinatus tendon was removed from the rotator cuff and then repaired in both shoulders. In one shoulder, a woven poly-L-lactide device was placed over the repair. In the other shoulder, the repair was left unaugmented. The augmented rotator cuff repair resulted in fewer tendon retractions, greater strength, and increased stiffness when compared to the contralateral untreated rotator cuff repairs. A recent study demonstrates that the application of the X-Repair device significantly increased the yield load and ultimate load of rotator cuff repairs in a human cadaveric model and altered the failure mode but did not affect initial repair stiffness [26].

## 4. Tissue Engineering with Mesenchymal Stem Cells (MSCs)

Technological advances in biology and engineering have resulted in marked improvements in the design and manufacture of tissue-engineered substitutes that can modify and maintain living tissue [27, 28]. Tissue engineering is an emerging field made up of the combination of scaffold, cell and stimulation or their stand-alone application [29, 30]. MSCs are capable of differentiating into a variety of specialized mesenchymal tissues including bone, tendon, cartilage, muscle, ligament, fat, and marrow stroma [29, 30]. Tissue engineering can be divided into two subtypes: the *in vivo* approach and the *ex vivo, de novo* one [27, 28]. The *in vivo* approach permits the self-regeneration of small tissue lesions. The *ex vivo, de novo* approach is designed to produce functional tissue that can be implanted in the body [31]. Tissue engineering is a multidisciplinary field founded on three fundamental principles: (I) the use of healthy multipotent cells that are nonimmunogenic, easy to isolate, and highly responsive to distinct environmental cues, (II) the development of carrier scaffolds that provide short-term mechanical stability of the transplant and a template for spatial growth of the regenerate tissue, and (III) the delivery of growth factors that drive the process of cell differentiation and maturation [27, 28]. MSCs can be applied directly to the site of injury or can be delivered on a suitable carrier matrix, which functions as a scaffold while tissue repair takes place [32]. 

The ideal scaffold for tendon engineering would possess the basic structure of the tendon, native extracellular matrix, and capability of cell seeding [33]. Decellularized multilayer tendon slices were seeded with BMSC, harvesting BMSC and infraspinatus tendons from dogs. Histology showed the alignment of the seeded cells between the collagen fibers of the tendon slices. qRT-PCR analysis showed higher tenomodulin and MMP13 expression and lower collagen type I expression in the composite than in the BMSC before seeding, suggesting that BMSC might express a tendon phenotype in this environment [33]. Delivering MSC-contracted, organized collagen implants applied to large tendon defects can significantly improve the biomechanics, structure, and probably the function of the tendon after injury [34]. A tissue prosthesis made up of cultured, autologous, marrow-derived MSCs suspended in a collagen gel delivery vehicle and contracted onto a pretensioned suture was implanted into a 1-cm-long gap defect in a rabbit Achilles tendon [34]. Load-related structural and material properties evaluated 4, 8, and 12 weeks later were greater than in the control repairs, which contained suture alone with natural cell recruitment. Furthermore, the treated tissue showed a significantly larger cross-sectional area, and their collagen fibers appeared to be better aligned than those in the controls. The use of MSCs to enhance allograft osteointegration is a novel method offering the potential of more physiologic and earlier healing [35]. MSCs derived from synovium have a higher proliferation and differentiation potential than the other MSCs. Their potential to accelerate the early remodelling of tendon-bone healing histologically by producing more collagen fibers at 1 week and forming more oblique collagen fibers connecting the bone to tendon resembling Sharpey's fibers at 2 weeks has been shown [36]. Moreover, MSCs do not interfere with tendon-bone healing at 4 weeks [36]. MSCs have been investigated in the management of tendinopathy. MSCs and IGF-I genes enhanced MSCs (AdIGF-MSCs) on the healing of a collagenase-induced bilateral tendinopathy lesions in an equine flexor digitorum superficialis injury model. Both MSC and AdIGF-MSC injections resulted in significantly improved tendon histological scores [37].

Tissue engineering techniques using novel scaffold materials offer potential alternatives for managing irreparable rotator cuff tears. A chitosan-based hyaluronan hybrid scaffold, with seeded fibroblasts to repair infraspinatus tendons defects produced in rabbits, demonstrated an enhanced type I collagen production and a significant improvement in tensile strength and tangent modulus from 4 to 12 weeks postoperatively [38]. *In vivo*, the effect of auto-crosslinked HA gel on adhesions and healing of injured and surgically repaired rabbit digital flexor tendons was studied, demonstrating a significantly faster increase in breaking strength with an accelerated tissue repair response after injury, but unaffected adhesions formation [39]. In rabbits, MSCs expanded in culture, suspended in type I collagen gel, and implanted into a surgically induced defect in the donor's right patellar tendon demonstrated significant increases in maximum stress, modulus, and strain energy density [40]. Changes in nuclear morphology of the MSCs in response to physical constraints provided by the contracted collagen fibrils may trigger differentiation pathways toward the fibroblastic lineage and influence the cell synthetic activity [41]. Controlling the contraction and organization of the cells and matrix will be critical to successfully produce tissue-engineered grafts. Seeded collagen gels with rabbit bone-marrow-derived MSCs and contracted onto sutures were implanted into full thickness, full length, central defects in the patellar tendons of the animals [40]. Repair tissues containing the MSC-collagen composites showed significantly higher maximum stresses and moduli than natural repair tissues at 12 and 26 weeks post surgery [40]. Autogenous tissue-engineered constructs were fabricated in culture between posts in the wells of silicone dishes [42]. Constructs were implanted in bilateral 2-cm-long gap defects in the rabbit's lateral Achilles tendon. At 12 weeks after surgery, no significant improvement was observed in any structural or mechanical properties or in histological appearance compared with control. The same authors tried also to determine how a tensile stimulus affects the gene expression of stem cell-collagen sponge constructs used to repair rabbit central patellar tendon defects [42]. MSCs were introduced into a gel-sponge composite showing cellular alignment comparable with that of normal tendon [43]. Cao et al. [4] tested the feasibility of engineering tendon tissues with autologous tenocytes to bridge a tendon defect in either a tendon sheath open model or a partial open model in the hen. FDP defects were bridged either with a cell-scaffold construct in the experimental group or with scaffold material alone in the control group. At 14 weeks, the engineered tendons resembled the natural tendons grossly in both colour and texture and displayed a typical tendon structure hardly distinguishable from that of normal tendons. The same authors also explored the feasibility of *in vitro *tendon engineering using the same type of cells and scaffold material [44]. Unwoven polyglycolic acid (PGA) fibers were arranged into a cord-like construct and fixed on a U-shape spring, and tenocytes were then seeded on PGA fibers to generate a cell-PGA construct. The results showed that tendon tissue could be generated during *in vitro* culture. In addition, the tissue structure and mechanical property became more mature and stronger with the increase of culture time. Alginate-based chitosan hybrid polymer fibers showed much improved adhesion capacity with tenocytes compared with alginate polymer fiber [45]. The rAAV-Gdf5 vector significantly accelerates wound healing in an *in vitro* fibroblast scratch model and, when loaded onto freeze-dried flexor digitorum longus tendon allografts, improves the metatarsophalangeal joint flexion to a significantly greater extent than the rAAVlacZ controls do [46]. In an experimental study on rabbits, a sharp complete midsubstance transection of the Achilles tendon was immediately repaired using a modified Kessler's suture and a running epitendinous suture. Both limbs were used, and each side was randomized to receive either bone-marrow-derived MSCs in a fibrin carrier or fibrin carrier alone (control). At 6 and 12 weeks, there were no differences between the groups with regard to morphometric nuclear parameters. Biomechanical testing showed improved modulus in the treatment group as compared with the control group at 3 weeks, but not at subsequent time periods [47]. 

Costa et al. [48] tried to optimize tenocyte proliferation in three tendon cell populations using growth factor supplementation. They isolated cells of the synovial sheath, epitenon, and endotenon from rabbit FDP tendons and maintained them in culture. For all three tendon cell populations, proliferation at 72 hours was greater in the presence of individual growth factors as compared with controls. In addition, a synergistic effect was observed. The combination of growth factors resulted in greater proliferation as compared with maximal doses of individual growth factors. Synthetic oligo[poly(ethylene glycol)fumarate]-(OPF-) based biomaterials were tested as a mean to deliver fibroblasts to promote regeneration of central/partial defects in tendons and ligaments. To further modulate the swelling and degradative characteristics of OPF-based hydrogels, OPF crosslinking via a radically initiated, mixedmode reaction involving poly(ethylene glycol) (PEG) diacrylate (PEG-DA) and PEG-dithiol was investigated. After encapsulation, tendon/ligament fibroblasts remained largely viable over 8 days of static culture. Although the presence of PEG-dithiol did not significantly affect cellularity or collagen production within the constructs over this time period, image analysis revealed that the 20% PEG-dithiol gels did appear to promote cell clustering, with greater values for aggregate area observed by day [49].

The use of a PEG-DA hydrogel incorporated with hydroxyapatite (HA) and the cell-adhesion peptide RGD (Arg-Gly-Asp) was tested as a material for determining an *in vitro* tissue interface to engineer intact ligaments. Incorporation of HA into PEG hydrogels reduced the swelling ratio but increased mechanical strength and stiffness of the hydrogels. Further, HA addition increased the capacity for cell growth and interface formation. RGD incorporation increased the swelling ratio but decreased mechanical strength and stiffness of the material [50].

 A novel fabrication system for photopatterning and assembling cell-laden OPF : PEG-DA hydrogels with high spatial fidelity and thickness using a controlled, inert nitrogen environment was described [51]. Cross-linking was performed using Irgacure-2959 photoinitiator and 365 nm light (7 mW/cm^2^) to form gels ranging from 0.9 to 3 mm in width. Employing an N^2^ environment increased gel thickness up to 240%, generating gels greater than 1 mm thick prior to swelling. This technique was further applied for spatially controlled patterning of primary tendon/ligament fibroblasts and marrow stromal cells in a single 1.5-mm-thick laminated hydrogel construct. Cells encapsulated using this technique maintained viability over 14 days in culture [51]. 

## 5. Discussion

The emerging field of tissue engineering holds the promise to use materials in tendon injury repair, namely, artificial polymers, biodegradable films, and biomaterials derived from animals or human (ECM devices) [7]. The most innovative strategy in tendon injury repair is the use of ECM matrices [52–62]. In contrast to traditional polymeric and metallic orthopaedic devices, intended to restore mechanical function and remain unchanged for the life of the patient [63–76], ECMs are temporary scaffold aimed to enhance and accelerate the biology of tissue repair [77, 78]. They undergo host cell infiltration and constructive tissue remodelling at variable rates [79]. Potential advantages of the use of ECM grafts include the capability to decrease the *in vivo* mechanical forces on the tendon repair during postoperative healing, to prevent repair gap formation or failure, to allow host cell infiltration and ideally even enhance the biology healing, and to be replaced by organized host tissue over time [80–87]. Additional research studies are required to verify these issues. The ideal scaffold should induce host-tissue ingrowth and tendon regeneration during the process of degradation, which varies dramatically among the commercially available scaffolds [88]. The capability of inducing host-tissue ingrowth is superior when using biological scaffolds, even though this process appears uncontrolled and nonspecific [89]. The interaction between scaffold surface and host cells is a key aspect of the use of scaffolds for tendon reconstruction. In the first phase of cellular ingrowth, multiple attachment points are established by the cells through the interaction between transmembrane proteins and proteins at the scaffold surface [6], later strengthened by accumulating integrin receptors, eventually forming a focal adhesion which acts as a connection between the actin cytoskeleton of the cell and the surface [6]. The cell proliferation cycle and cell migration start after the formation of focal adhesions and spreading of cells on the surface [6]. Cell attachment, proliferation, and migration is facilitated by the porosity of scaffolds [90]. The surface of biological scaffolds is mostly composed of natural type I collagen protein, which determines a higher affinity to host cells and, therefore, promotes cellular adhesion, proliferation, migration, and tissue induction [6]. 

On the other hand, the surfaces of synthetic scaffold are composed of macromolecules lacking a well-defined structure that allows host cell to produce a strong binding point and start growing [6].

Even though biological scaffolds are becoming more popular, clinical well-conducted human studies are lacking, and little data describing the complications or adverse events associated with the use of these products are available. ECMs fabricating in parallel with other materials may increase their mechanical properties, such as natural ECMs seeded with bone marrow stem cells or tenocytes [91–101]. However, clinical evidence in this field is scanty. Major concern about both biological and synthetic scaffolds is the biocompatibility and the inflammatory response associated with foreign body rejection [6]. To decrease the bioburden and the risk of inflammatory or foreign body reactions, all tissues, regardless of their origin, are extensively purified to remove proteins, cells, and lipids. Some graft options have been artificially crosslinked to decrease antigenicity, by decreasing their sensitivity to collagenases [1, 102–113]. Although rare, aseptic, nonspecific inflammatory reactions and foreign body-like reactions have been reported with certain xenografts [7, 8, 12, 114, 115]. Aseptic reactions were reported in 16–22% [115] of implantations, always with negative aspirates and cultures, destroyed xenografts, and histopathological evidence of inflamed granulation tissue with abundant neutrophils, but no foreign body reaction, as documented by the absence of organisms, crystals, or giant cells [8, 115, 116]. Valentin et al. [88] examined the host-tissue morphologic response to five commercially available extracellular matrix-derived biological scaffolds (GraftJacket, Restore, CuffPatch, TissueMend, Permacol) used for orthopaedic soft-tissue repair in a rodent model. Each device elicited a distinct morphologic response that differed with respect to cellularity, vascularity, the presence of multinucleated giant cells, and organization of the remodelled tissue [117–129]. More rapidly degraded devices such as Restore and autologous tissue showed the greatest amount of cellular infiltration, especially at the early time points [78, 130–146]. Devices that degraded slowly, such as CuffPatch, TissueMend, and Permacol, were associated with the presence of foreign-body giant cells, chronic inflammation, and/or the accumulation of dense, poorly organized fibrous tissue [2, 147–160]. Depending on the product, processing may involve acellularization treatment, chemical cross-linking, lamination of multiple layers or lyophilization [161]. These biomaterials have incomplete acellularization [12, 77], and the clinical implications are still not clear. Acellularization treatment aims to reduce antigenicity, by disrupting cells and removing water-soluble cellular proteins. Acellularization may also enhance host cell infiltration with phenotypically appropriate cells [162] and possibly prevent transmission of infectious genomic vectors [163]. Further biochemical and immunologic investigations are required to establish whether and how much acellularization treatment increases the safety and efficacy of these implants. The use of  biological scaffolds manufactured from human or animal tissue carries also the risk of disease transmission, which, even though not reported to date, remains a theoretical concern [164–176]. Obviously, there is no risk of disease transmission with the use of synthetic scaffolds [6]. One of the advantages of biomaterials is that exogenous growth factors, gene therapy approaches, or cell delivery can be used together with these biomaterials. Several chemical cross-linking agents (i.e., glutaraldehyde, polyepoxy compound, carbodiimide, genipin, isocyanate, and proanthocyanidin) have been used to stabilize the collagen structure of the scaffold, maintaining the mechanical properties [176–189]. Clinical studies have not confirmed the expected beneficial effect of chemical cross-linking scaffolds. Further investigations are warranted to establish the *in vivo* benefit of chemical cross-linking in biocompatibility and mechanical properties on the scaffolds. As Chen et al. [6] proposed, another reason of concern is that available scaffolds are produced to mimic the tendon or ligament extracellular microenvironment to stimulate cell proliferation and tissue ingrowth, largely ignoring the healing process at the enthesis. The repair procedure often involves reconstruction of the junction, and failure of surgery is frequently caused by osteolysis and scaffold pullout. Further investigations are required to better understand how to promote the healing of bone-tendon junction. 

## 6. Conclusion

Tendon disorders are frequent and cause significant morbidity both in sports and workplace [144, 177, 190]. Several conservative and surgical procedures are available for tendon healing, but one of the major problems encountered when dealing with tendinopathies is that etiology is largely unknown [65, 86, 94, 96, 173]. 

Preliminary studies support the idea that scaffolds can provide an alternative for tendon augmentation with an enormous therapeutic potential. However, available data are lacking to allow definitive conclusion on the use of scaffolds for tendon augmentation. Additionally, the prevalence of postoperative complications encountered with their use varies within the different studies. Further investigations are required to evaluate the role of scaffolds in the clinical practice.
